# Brain Tumor Segmentation via Multi-Modalities Interactive Feature Learning

**DOI:** 10.3389/fmed.2021.653925

**Published:** 2021-05-13

**Authors:** Bo Wang, Jingyi Yang, Hong Peng, Jingyang Ai, Lihua An, Bo Yang, Zheng You, Lin Ma

**Affiliations:** ^1^The State Key Laboratory of Precision Measurement Technology and Instruments, Department of Precision Instrument, Tsinghua University, Beijing, China; ^2^Beijing Jingzhen Medical Technology Ltd., Beijing, China; ^3^School of Artificial Intelligence, Xidian University, Xi'an, China; ^4^Department of Radiology, The 1st Medical Center, Chinese PLA General Hospital, Beijing, China; ^5^Radiology Department, Affiliated Hospital of Jining Medical University, Jining, China; ^6^China Institute of Marine Technology & Economy, Beijing, China

**Keywords:** brain tumor segmentation, deep neural network, multi-modality learning, feature fusion, attention mechanism

## Abstract

Automatic segmentation of brain tumors from multi-modalities magnetic resonance image data has the potential to enable preoperative planning and intraoperative volume measurement. Recent advances in deep convolutional neural network technology have opened up an opportunity to achieve end-to-end segmenting the brain tumor areas. However, the medical image data used in brain tumor segmentation are relatively scarce and the appearance of brain tumors is varied, so that it is difficult to find a learnable pattern to directly describe tumor regions. In this paper, we propose a novel cross-modalities interactive feature learning framework to segment brain tumors from the multi-modalities data. The core idea is that the multi-modality MR data contain rich patterns of the normal brain regions, which can be easily captured and can be potentially used to detect the non-normal brain regions, i.e., brain tumor regions. The proposed multi-modalities interactive feature learning framework consists of two modules: cross-modality feature extracting module and attention guided feature fusing module, which aim at exploring the rich patterns cross multi-modalities and guiding the interacting and the fusing process for the rich features from different modalities. Comprehensive experiments are conducted on the BraTS 2018 benchmark, which show that the proposed cross-modality feature learning framework can effectively improve the brain tumor segmentation performance when compared with the baseline methods and state-of-the-art methods.

## 1. Introduction

Brain cancer is an aggressive and highly lethal malignancy that has received more and more attention and presented multiple technical challenges for studies on brain tumors. Owing to the diversity of the appearance and morphology of brain tumors, accurately automatically segmenting tumor areas from multi-modality magnetic resonance image (MRI) sequences is a difficult but meaningful issue in field of artificial intelligence and assisted diagnosis (1). In this paper, we study a deep-learning based automatic brain tumor segmentation network to assist clinicians in improving the diagnostic efficiency of brain tumors. For the automatically tumor segmentation task, the input medical images are multi-modality data and the corresponding segmentation masks contain multi areas of the brain tumor. Specifically, the input multi-modality medical image consist of four MRI modality, i.e., T1-weighted (T1) modality, contrast enhanced T1-weighted (T1c) modality, T2-weighted (T2) modality, and T2 Fluid Attenuation Inversion Recovery (FLAIR) modality. The goal of brain tumor segmentation is to determine the volume, shape, and localization of brain tumor areas, i.e., the whole tumor (WT) area, the tumor core (TC) area, and the enhancing tumor (ET) core area, which play crucial roles in brain tumor diagnosis and monitoring.

To achieve automatic brain tumor segmentation, some methods use the deep convolutional neural network (DCNNs) to extract the features of tumors and determine the labels of multi-class pixels in the end-to-end fashion. However, existing brain tumor segmentation methods (2–4) usually consider this task as a semantic segmentation problem for common nature images, which methods omit the great disparity between the medical image and the common nature image. Specifically, there are two-fold distinct properties between these two kinds of images: (1) As a departure from the common nature image, the medical image usually consist of multiple MRI modalities that capture different pathological properties. (2) The geometrical shape, spatial position, and texture structure of tumor in medical images are complex and changeable, and the tumor does not have a specific, regular pattern of appearance. Therefore, such existing approaches would not obtain the optimal solutions.

Due to the above discussions properties, for the brain tumor segmentation task, the deep learning based segmentation methods still has challenging issues needed to be addressed. First, the existing methods cannot fully mine the potential knowledge in multi-modalities. Specifically, the previous works use simple parameter-sharing feature extractors to obtain features of different modal data and directly concatenate the information from different modality data. Such feature extraction and processing methods lack a data mining strategy for effectively informational fusing and extracting knowledge from complex data structures. Second, due to the nonspecific structural pattern in the tumor area, the existing supervised learning-based segmentation methods, which are guided only by a manually annotated foreground and background segmentation ground truth, are difficult to learn the complete discriminant information of brain tumor.

To address these issues, in this paper, we proposed a novel interactive modality deep feature learning framework to learn the discriminant information of brain tumor from the multi-modality MRI data. Considering the fact that the texture and spatial position of normal organs in medical images have specific structural patterns, and deep neural networks can easily learn discriminant information from such regular patterns. Meanwhile, radiologists need to combine information from multiple modalities to determine the full range of areas of a brain tumor. For the multi-modality MRI data, the intra-modality information describes the discriminant feature between the normal organ and the lesion area (i.e., brain area and tumor area) in medical images, the inter-modality information provides additional cross-modal constraints for determining the visual boundaries and different regions of the brain tumor. Specifically, the proposed interactive modality deep feature learning framework consists of the cross-modality feature extraction and the normal region-guided feature fusion.

**Figure 1:**
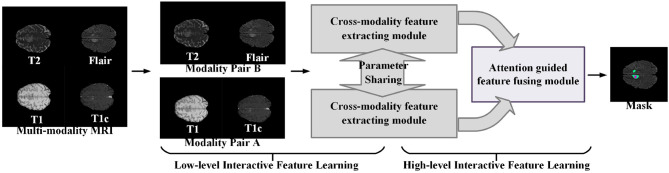
A brief illustration of the proposed multi-modality interactive feature learning framework for brain tumor segmentation.

Figure 1 illustrates the proposed learning framework briefly. In the cross-modality feature extracting process, we adopt a two-step feature interacting strategy to extract the interactive features across different modality data. The first feature interacting step concatenates multi-modality image data in channel-wise to extract the low-level interactive features at input level, and the second feature interacting step integrates the high-level features of different modality pairs to extract the high-level interactive features. In the normal region-guided feature fusion, we propose a novel reverse attention-based feature fusion framework to collectively enhance the features of normal brain region from different modality data. This encourages the feature extracting network to learn intrinsic patterns that are helpful to determine the normal brain area from each modality data. The intuition behind this process is that the reverse attention mechanism enhance the non-tumor regions in the brain MRI data, and those regions contain rich structure and texture information of normal brain regions.

## 2. Related Works

### 2.1. Brain Tumor Segmentation

Brain tumor segmentation is a hot topic in the medical image analysis and machine learning community. It has received great attention in the past few years. Early efforts in this filed designed hand-crafted features and adopted the classic machine learning models to predict the brain tumor areas. Due to the rapid development of the deep learning technique (5–9), the recent brain tumor segmentation approaches mainly apply the deep features and classifiers from the DCNN models. Based on the type of the convolutional operation used in the DCNN models, we briefly divide the existing methods into two groups, i.e., the 2D CNN-based methods and 3D CNN-based method. The 2D CNN-based methods (10–12) apply the 2D convolutional operations and split the 3D volume samples into 2D slices or 2D patches. While the 3D CNN-based methods (13–16) apply the 3D convolutional operations, which can take the whole 3D volume samples or the extracted sub-3D patches as the network input.

### 2.2. Multi-Modality Feature Learning

Multi-modality feature learning is gaining more and more attention in the recent years as the multi-modality data can provide richer information for sensing the physical world. Existing works have applied multi-modality feature learning in many computer vision-based tasks such as 3D shape recognition (17–20) and retrieval (21–24), survival prediction (25), RGB-D object recognition (26), and person re-identification (27). Among these methods, Bu et al. (21) built a multi-modality fusion head to fuse the deep features learnt by a CNN network branch and a deep belief network (DBN) branch. To integrate multiple modalities and eliminate view variations, Yao et al. (25) designed a deep correlational learning module for learning informative features on the pathological data and the molecular data. Wang et al. (28) proposed a large-margin multi-modal deep learning framework to discover the most discriminative features for each modality and harness the complementary relationship between different modalities.

## 3. Dataset Description

We implement all experiments on BraTS 2018 benchmark (29–31) to evaluate the performance of proposed brain tumor segmentation. The BraTS 2018 benchmark dataset contains four modalities, i.e., T1, T1-c, T2, and FLAIR, for each patient. The BraTS 2018 benchmark has two subsets: a training set, which contains 285 subjects, and a validation set containing 66 subjects with hidden ground truth. Each subject holds a manual expert segmentation of three tumor sub-compartments: edema (ED), ET, and necrotic tissue combined with non-enhancing tumor (NCR/NET). In the official BraTS evaluation, these sub-compartments are combined into three hierarchical labels: WT, TC, and ET. WT is a combination of all tumor sub-compartments (i.e., ET, NCR/NET), TC combines ET and NCR/NET, and ET is defined by the ET sub-compartment. Aiming at yielding uncertainty estimates for these hierarchical tumor regions, we combined the tumor sub-compartment labels into the hierarchical labels before the training of the automated segmentation models. The BraTS 2018 dataset comes preprocessed; the subjects and MR images are co-registered to the same anatomical template, resampled to unit voxel size (1 × 1 × 1), and skull stripped. When implementing the experiments on each of the benchmarks, we randomly select the 80% data in training set to train the brain tumor segmentation models while use the rest of the data in training set to test the segmentation performance. We additionally normalized each MR image subject-wise to zero mean and unit variance.

## 4. Methods

The aim is to segment the brain tumor regions including the WT region, the TC region, and the enhancing TC region from multi-modality MRI data. For this purpose, we propose to build a multi-modality-based single prediction multi-region segmentation method that utilizes the cross-modalities interactive features from MRI data. In this work, we propose to train a cross-modalities interactive feature extracting and fusing network using reverse attention guidance and use the trained network for segmenting brain tumor regions in MRI data.

In this section, we first describe the network architecture and the workflow of the proposed multi-modalities brain tumor segmentation framework and also including the details of the cross-modality feature extracting process and the attention-guided feature fusion that are two important interactive feature learning modules. Then, we introduce the implement details of the training process and experiments.

### 4.1. Multi-Modalities Brain Tumor Segmentation Network

**Figure 2:**
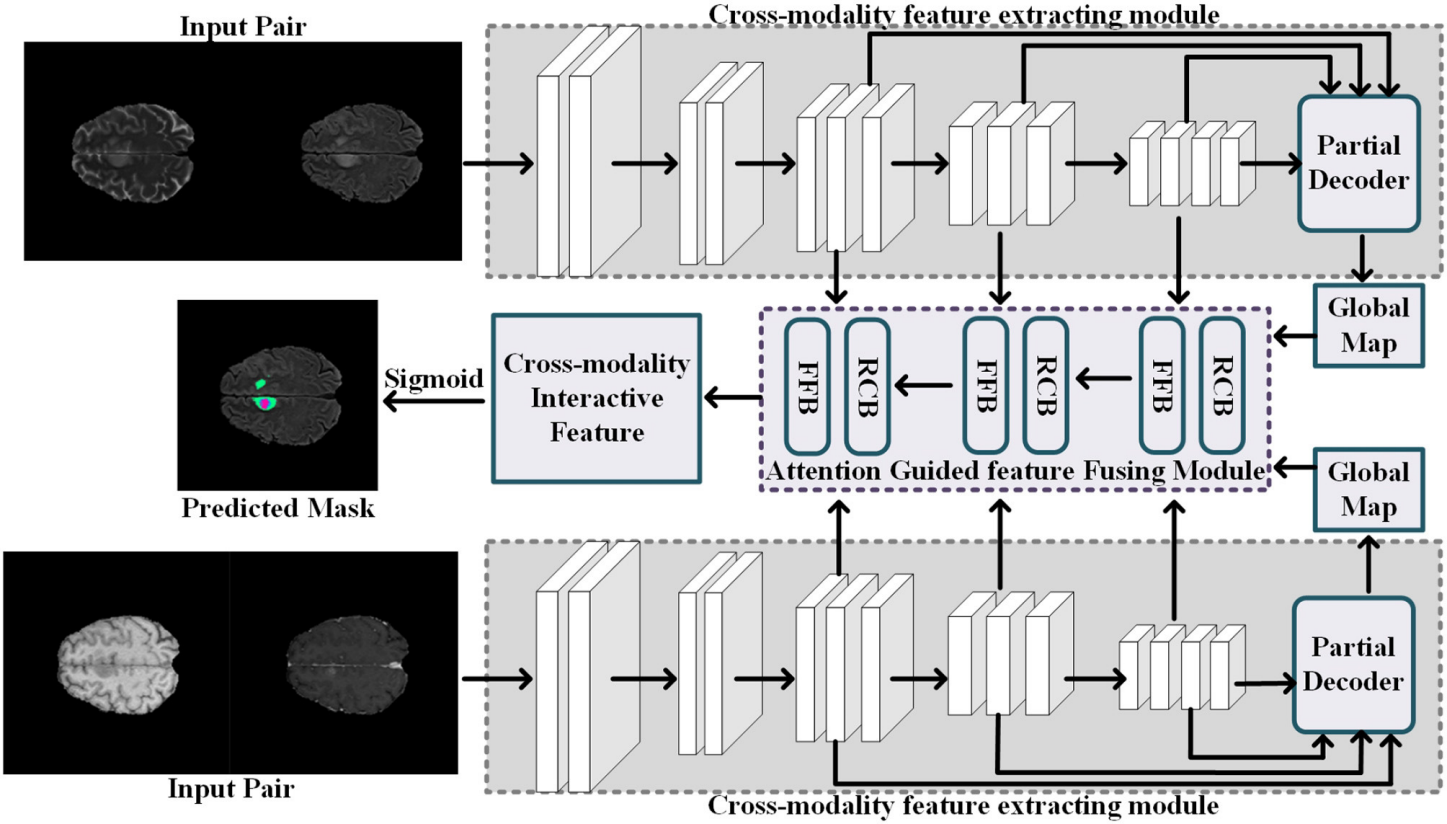
Illustration of the network architecture of the segmentation process.

Given an input MRI data **X** = {*x*_*T*1_, *x*_*T*1*c*_, *x*_*T*2_, *x*_*FLAIR*_}, where the variables *x*_*T*1_,*x*_*T*1*c*_,*x*_*T*2_, and *x*_*FLAIR*_ represent the T1-weighted modality, the contrast-enhanced T1-weighted modality, the T2-weighted modality, and the fluid attenuation inversion recovery modality, respectively, we follow the work (32) to split the multi-modalities input **X** to form two modality pairs *X*_*g*1_ = {*x*_*T*1_, *x*_*T*1*c*_} and *X*_*g*2_ = {*x*_*T*2_, *x*_*FLAIR*_}, which encourages the information within each modality pair tends to be consistent while the information from different modality pairs tends to be distinct and complementary. The cross-modality feature extracting module takes the modality pair as input, and outputs the interactive features of the multi-modalities data. Then, the attention-guided feature fusion module takes the interactive features as input and output the fused cross-modality interactive feature. Finally, the segmentation results of the brain tumor region are generated from the fused cross-modality interactive feature. The network architecture of our proposed multi-modalities brain tumor segmentation framework is shown in Figure 2. Each component will be elaborated as follows.

#### 4.1.1. Cross-Modality Feature Extracting Module

Current popular multi-modalities feature extracting network usually rely on a single simple interactive strategy, i.e., the channel concatenation (33) or the parameters sharing (34). The channel concatenation strategy only considers the common features among different modalities, but ignore the richness of the features brought by the modes; conversely, the parameters sharing strategy only pays attention to the richness of features brought by multi-modalities, but ignores the common features among different modalities. To effectively interact features between different modalities, we employ the combinational strategy of both the channel concatenation and the parameters sharing to extract the common features among similar modalities and use the information between different modalities to improve the richness of the features. Specifically, we use a CNN-based network to extract the common features in a modality pair where the modalities sharing consistent feature for common pathological areas and normal areas, as shown in Figure 2. The cross-modality feature extracting module has two input channels corresponding to the two MR images from one modality pair, i.e., *X*_*g*1_ = {*x*_*T*1_, *x*_*T*1*c*_} or *X*_*g*2_ = {*x*_*T*2_, *x*_*FLAIR*_}. Meanwhile, the feature extractor is sharing parameters for extracting the interactive features of the different modality pairs.

Considering the low-level features contribute less to segmentation performance but demand more computational resources, we aggregate the high-level features to predict the common brain tumor areas in each modality pair. Specifically, for an input modality pair *x*_*g*1_ = {*x*_*T*1_, *x*_*T*1*c*_} (or *x*_*g*2_ = {*x*_*T*2_, *x*_*FLAIR*_), each modality data with size *h* × *w* × *l*, five levels of features *f*_*i*_, *i* = 1, …, 5 with resolution [*h*/2^*k*−1^, *w*/2^*k*−1^, *l*/2^*k*−1^] can be extracted from the cross-modality feature extracting network. Then, we follow the work (35) to divide interactive features *f*_*i*_ into low-level features group {*f*_*i*_, *i* = 1, 2} and high-level features group {*f*_*i*_, *i* = 3, 4, 5}. The low-level features contain lots of modality information, which are not applicable to interactive features fusion between multi-modalities. Thus, we employ the partial decoder *D*_*p*_ (35) to only aggregate the high-level feature {*f*_*i*_, *i* = 3, 4, 5} with a cascade fashion. The interactive feature of one modality pair is computed by the *f*_*Dp*_ = *D*_*p*_(*f*_3_, *f*_4_, *f*_5_), and we also can obtain the global map *M*_*g*_ of the input modality pair.

#### 4.1.2. Attention Guided Feature Fusing Module

The global map *M*_*g*_ is formed by the high-level features {*f*_*i*_, *i* = 3, 4, 5}, which captures the high-level information such as normal brain areas and tumor areas. However, the rich diversity of brain tumor regions makes it impossible for feature extraction models to extract a learnable structural pattern from this region. Compared with brain tumor regions, the normal brain regions in the training images are regularly distributed, and these structural patterns are easier to perceive and extract. Motivated by this observation, we propose a cross-modality features fusing strategy to progressively discriminative brain regions through an erasing foreground object manner [pranet 27,4]. Instead of predicting the non-normal regions (brain tumor areas) directly, we propose to determine the normal brain regions in the multi-modalities MR data by learning the reverse attention (35) from the high-level features. The proposed attention-guided feature fusing module consists of two blocks: the feature fusion block and the reverse co-attention block.

**Figure 3:**
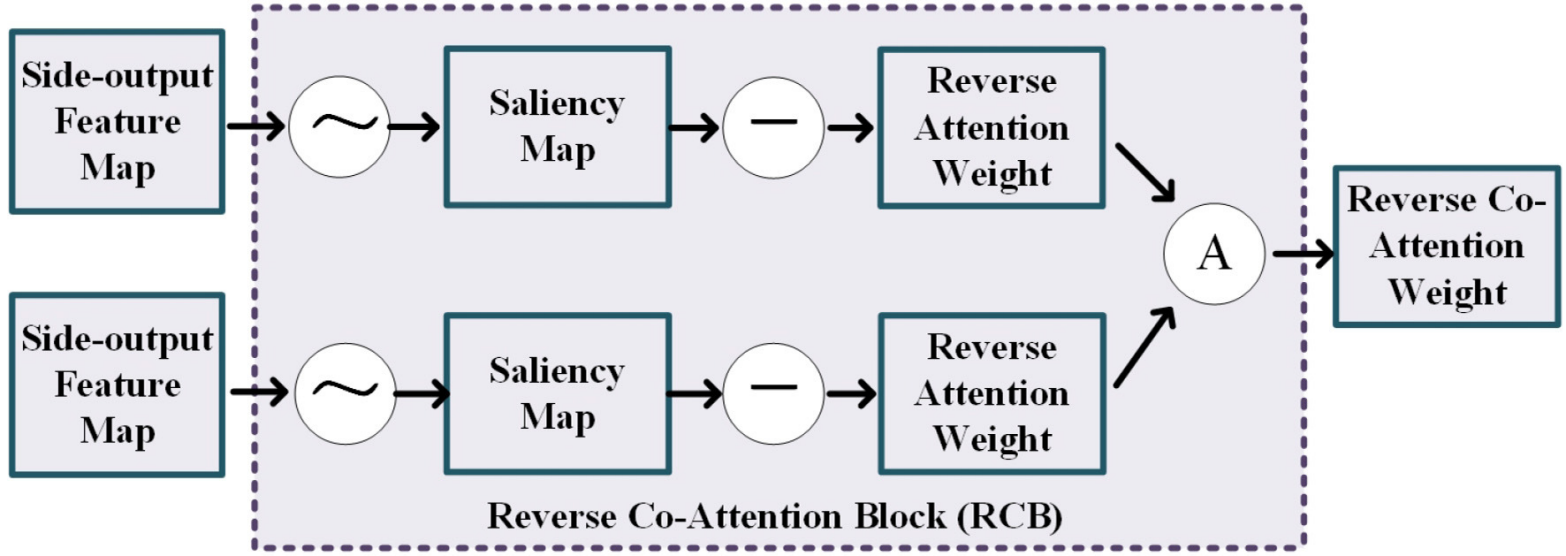
Illustration of the details of the reverse co-attention block, where the “A” represents the average operation.

As shown in Figure 3, the reverse co-attention block takes two side-output feature maps from two modality pairs as input and outputs a reverse co-attention weight. The side-output feature maps *M*_*i*_, *i* = 3, 4, 5 are generated by the previous FFD (feature fusing block). In each reverse co-attention block, a sigmoid operation and a reverse operation are used to generate the reverse attention weight *R*_*i*_. The reverse attention weight *R*_*i*_ is a negative salient object detection in the computer vision community (36–39) and can be formulated as Equation (1):

(1) $$\begin{array}{r} R_{i}=⊝\left(\sigma \left(M_{i}\right)\right) \end{array}$$

where the ⊝ denotes a reverse operation subtracting the input from all 1's matrix **E** and σ is the Sigmoid function. To explore the high-level interactive features of the two modality pairs, we average the reverse attention weights from the two cross-modalities feature extracting module to generate a reverse co-attention weights ${\bar{R}}_{i}$.

**Figure 4:**
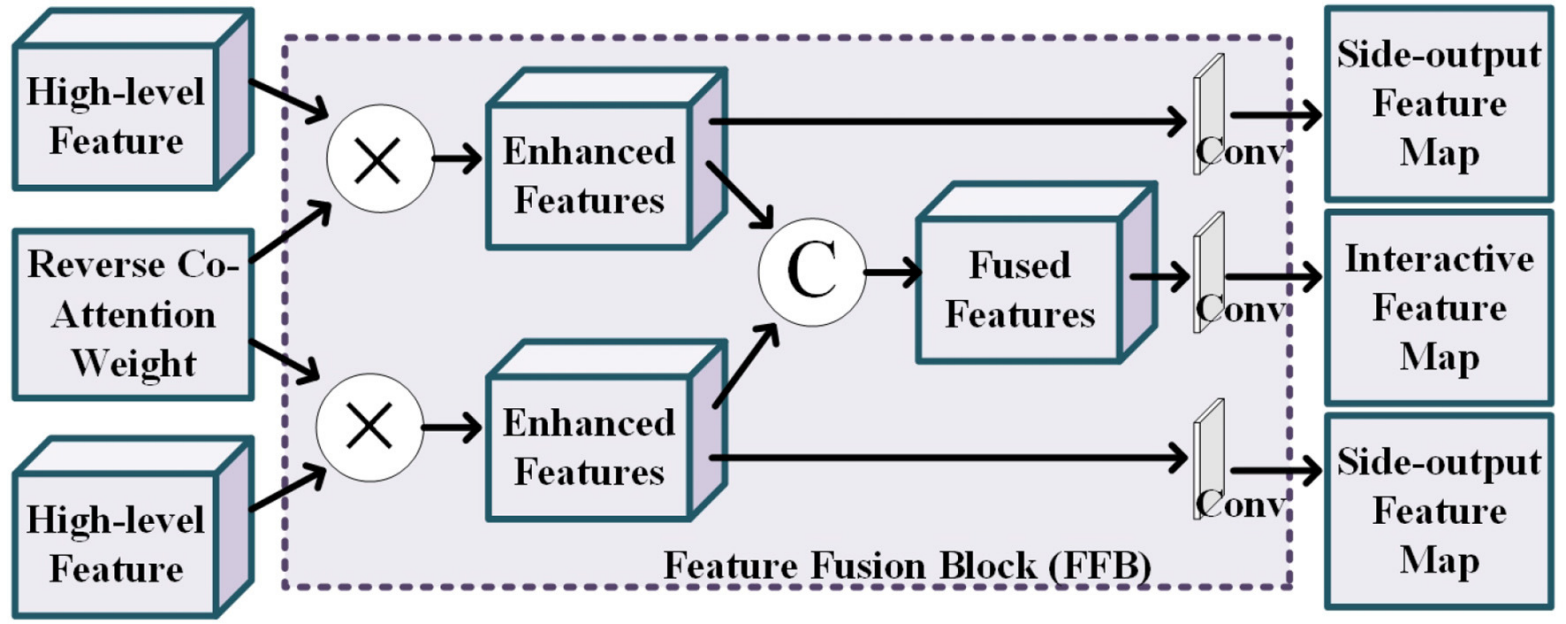
Illustration of the details of the feature fusion block, where the operation “C” represents the channel-wise concatenation.

The details of feature fusing block is shown in Figure 4. This block tasks the high-level features of the two modality pairs and a reverse co-attention weight as input to generate the side-output feature maps and the interactive feature map. The reverse co-attention weight enhances the features of the common interest regions in the two modality pairs, and weakens the features of the common no interest regions, which will enable deep integration of features between multiple modality pairs. Specifically, the output interactive features $\left\{{\bar{f}}_{i},i=3,4,5\right\}$ of each modality pair can be obtained by element-wise multiplying (⊗) the high-level feature {*f*_*i*_, *i* = 3, 4, 5} by the reverse co-attention weight ${\bar{R}}_{i}$, as Equation (2):

(2) $$\begin{array}{r} {\bar{f}}_{i}=f_{i}⊗{\bar{R}}_{i+1} \end{array}$$

We concatenate the reverse co-attention feature of the two modality pairs in channel-wise to deeply fuse the features of the two modality pairs. The final segmentation result is obtained by progressively superpose the fused features.

### 4.2. Learning Process and Implementation Details

#### 4.2.1. Loss Function

Our loss function consist of segmentation loss $L_{sg}$ and saliency detection loss $L_{sd}$. The $L_{sg}$ is Dice Similarity Coefficient (DSC) (32), which evaluates the similarity between two higher-dimensional sets, i.e., the segmentation masks and the ground-truth masks, and can be formulated as Equation (4):

(3) $$\begin{array}{r} L_{sg}\left(\text{Y},\text{U}\right)=1-\frac{2\times |\text{Y}⋂\text{S}|}{\left|\text{Y}|+|\text{S}\right|} \end{array}$$

where **Y** and **S** represent the ground-truth annotation and the segmentation mask for the desired brain tumor areas, respectively.

The saliency detection loss $L_{sd}$ implements deep supervision for the three side-output feature maps {*M*_3_, *M*_4_, *M*_5_} and the global map *M*_*g*_, which prevents the model from being heavily affected by the unbalance among different types of tumor areas. We adopt weighted binary cross entropy (BCE) loss to achieve this proposal. The weighted BCE loss pays more attention to hard pixels rather than assigning all pixels equal weights (35). The definitions of these losses are the same as in [21,26] and their effectiveness has been validated in the field of salient object detection. Each map is up-sampled $M_{i}^{up}$ to the same size as the ground-truth map **G**, which is obtained by dividing the tumor regions annotation into three separate binary maps (i.e., WT map, ET map, and TC map). The deep supervision loss *L*_*deep*_ can be formulated as Equation (4):

(4) $$\begin{array}{r} L_{deep}=L_{sd}\left(\text{G},M_{g}^{up}\right)+\overset{5}{\underset{i=3}{\sum }}L_{sd}\left(\text{G},M_{i}^{up}\right) \end{array}$$

The total loss function *L*_*total*_ can be formulated as Equation (5):

(5) $$\begin{array}{r} L_{total}=\alpha L_{sg}\left(\text{Y},\text{U}\right)+\left(1-\alpha \right)\left(L_{sd}\left(\text{G},M_{g}^{up}\right)+\overset{5}{\underset{i=3}{\sum }}L_{sd}\left(\text{G},M_{i}^{up}\right)\right) \end{array}$$

where the weight α is empirically set to 0.7.

#### 4.2.2. Implementation Details

We follow the work (32) to adopt the pre-trained parameters of transition generative networks to initialize the feature extracting network in our methods. Specifically, each of the input modality data was normalized to have zero mean and unit variance, and the inputs of both the cross-modality feature transition are randomly sampled from the training data set, and the input patch size is 128 × 128 × 128. We also employ U-net as backbone, where the base number of filters is 16 and increased to twice after each down-sampling layer. We use Adam optimizer with an initial learning rate is 10-e4 and λ is 10 to optimize the objective function. The network branches were implemented in Pytorch on four NVIDIA GTX 1080TI GPU. It totally takes 5 h to complete the training process and the test speed is 2.5 s per subject.

## 5. Evaluation Metrics

The performance of the segmentation algorithm is evaluated based on two metrics, i.e., the Dice score, and the 95th percentile of the Hausdorff Distance (Hausdorff95).

The Dice score is a commonly used metric for measuring the segmentation accuracy at the pixel level. It is a statistical gauge of the similarity between two sets of samples. Given *S*, a set of pixels belonging to a ground truth of the segmentation mask of brain tumor regions, and *P*, a set of pixels belonging to a predicted segmentation mask of the brain tumor regions.The Dice score is defined as in Equation (6), where |·| denotes set cardinality. The Dice score ranges from 0 (no overlap between *S* and *P*) to 1 (perfect overlap between *S* and *P*), and the lower is better.

(6) $$\begin{array}{r} Dice=\frac{2\times |\text{S}⋂\text{P}|}{\left|\text{S}|+|\text{P}\right|} \end{array}$$

The 95th percentile of the Hausdorff Distance (Hausdorff95) is a boundary-based segmentation accuracy evaluation metric. It calculates the distance between the two point sets. Considering the predicted segmentation mask *P* and the ground-truth mask *S*, the Hausdorff distance between the two set is defined as Equation (7):

(7) $$\begin{array}{r} d_{H}\left(S,P\right)=max\left[max_{p\in P}min_{s\in S}\left[D\left(S,P\right)\right],max_{s\in S}min_{p\in P}\left[d\left(S,P\right)\right]\right] \end{array}$$

where the *d*_*H*_(*x, y*) denotes the distance between pixels *x* ∈ *P* and *y* ∈ *S*. We follow the work (40) to use Euclidean distance to calculate the pixel-wise distance. The Hausdorff distance represents the longest distance from *P* (respectively *S*) to its closest point in *S* (respectively *P*). It is the most extreme value from all distances between the pairs of the nearest pixel on the boundaries of *S* and *P*. Finally, the score of Hausdorff distance is multiplied by 95% to eliminate the interference from outlier points.

In this work, the predicted segmentation masks are compared with the ground-truth masks via Dice score and the 95th percentile of Hausdorff distance (Hausdorff95). A higher Dice coefficient and a lower Hausdorff distance indicate the efficacy of the brain tumor segmentation method.

## 6. Results

This section presents quantitative and qualitative evaluations of the performance of the proposed segmentation method to segment the three brain tumor regions in the multi-modality MRI data.

### 6.1. Ablation Study of the Proposed Approach

For the analysis of the contribution and the effect of our proposed branches for brain tumor segmentation task, we conduct the experiments on the following baseline models. The first two baseline models train the single-modality-pair feature extracting modules “*f*_*g*1_” and “*f*_*g*2_” with the input modality data *X*_*g*1_ = {*x*_*T*1_, *x*_*T*1*c*_} or *X*_*g*2_ = {*x*_*T*2_, *x*_*FLAIR*_}, respectively. The third baseline model “*f*_*g*1+2_” fuses the prediction of “*f*_*g*1_” and “*f*_*g*2_” by directly computing the average of the obtained segmentation maps without using any feature fusing strategies proposed in this paper. The first three baselines are designed to analyze the contribution of the multi-modalities of the MRI data for segmenting the brain tumor regions. We also introduce two baseline models “Ours w AT” and “Ours w/o CA” to analyze the contribution of the proposed reverse attention-guided feature fusion and segmentation module. Specifically, “Ours w AT” represents the feature fusion module use, a saliency attention strategy (41) to fuse the cross-modalities features, and “Ours w/o CA” represents the feature fusion module use, the independent reverse attention that do not interact between the modality pairs to guide the feature fusing. We use the parameters of the pre-trained generative feature transition network (32) to initialize all the aforementioned baseline models, and these baselines are fine-tuned on the same training data as our method. The experimental results are reported in top rows of Table 1.

**Table 1:** Ablation study of the proposed approach and the other baseline models on the BraTS 2018 validation set.

**Methods**	**Dice score**				**Hausdorff95**			
	**WT**	**ET**	**TC**	**Average**	**WT**	**ET**	**TC**	**Average**
“*f*_*g*1_”	0.698	0.793	0.808	0.766	4.412	9.614	8.184	7.403
“*f*_*g*2_”	0.517	0.876	0.749	0.714	10.461	5.668	9.472	8.534
“*f*_*g*1+2_”	0.674	0.818	0.782	0.758	5.072	6.101	8.562	6.578
“Ours w/o CA”	0.778	0.885	0.819	0.827	3.841	5.912	7.291	5.681
“Ours w AT”	0.789	0.897	0.836	0.841	4.690	4.912	6.912	5.505
Ours	0.801	0.909	0.854	0.855	3.879	4.571	6.411	4.954

*Higher Dice scores indicate the better results, while lower Hausdorff95 scores indicate the better results*.

By comparing single-modality pair modules (“*f*_*g*1_” and “*f*_*g*2_”) and the multi-modality pair baseline “*f*_*g*1+2_”, we observe that the baseline achieves more stable performance than the single-modality pair modules, but it does not achieve the better comprehensive performance than baseline “*f*_*g*1_.” This can demonstrate that the arbitrary feature fusion has limited improvement on segmentation performance due to the lack of effective fusion strategy. By comparing the attention-guided feature fusion baselines (i.e., “Ours w/o CA” and “Ours w AT”) with the “*f*_*g*1+2_”, we can observe that the attention-guided feature fusion can improve the segmentation performance. It demonstrates that the performance improvement of our method mainly comes from the well-designed multi-modalities feature fusion and learning strategy. By comparing “Ours” with baselines “Ours w/o CA” and “Ours w AT,” we can observe that the common attention of modality pairs plays an important role in fusing informative features and predicting accurate tumor areas (see “Ours w/o CA” vs. “Ours w AT”), and the reverse attention mechanism can further improve the segmentation performance (see “Ours” vs. “Ours w AT”). The ablation analysis demonstrates the contribution of our proposed cross-modality feature extracting module and attention guided feature fusing module for improving the performance of brain tumor segmentation.

### 6.2. Comparison With State-of-the-Art Methods

To evaluate the effectiveness of the proposed brain tumor segmentation model, on the BraTs2018 dataset, we follow the work of (32) to compare the segmentation performance of the proposed method with seven state-of-the-art methods including three ensemble-models methods, i.e., Myronenko (33), Isensee et al.(42), Puch et al.(2), and four single-prediction methods: Chandra et al. (3), Ma et al. (4), Chen et al.(43), and Zhang et al. (32). The quantitative results are reported in Table 2. The performances of the segmentation models were evaluated with the Disc score and Hausdorff95. From Table 2, we can observe that our methods achieve the best performance when comparing with the state-of-the-art single-prediction methods both in terms of Dice score and Hausdorff95. When comparing with the ensemble-models methods, our method has the second best performance. Usually, the ensemble-models methods can usually obtain better performance than the single-prediction methods, since the ensemble models methods integrate multiple brain tumor segmentation models that are trained by using different views or different training subsets, while the single prediction methods only use one segmentation model to implement multi-brain tumor areas segmentation tasks. However, the ensemble-models methods require training multiple models with more training data, which means higher complexity both in computational cost and time consumption. Considering the balance between time cost and algorithm performance, the performance of our method is satisfactory. Thus, the comparison results in Table 2 demonstrate the effectiveness of the proposed approach.

**Table 2:** Comparison results of the proposed approach and the other state-of-the-art models on the BraTS 2018 validation set.

**Methods**	**Dice score**				**Hausdorff95**			
	**WT**	**ET**	**TC**	**Average**	**WT**	**ET**	**TC**	**Average**
Myronenko (33)	0.823	0.910	0.867	0.866	3.926	4.516	6.855	5.099
Isensee et al. (42)	0.809	0.913	0.863	0.861	2.410	4.270	6.520	4.400
Puch et al. (2)	0.758	0.895	0.774	0.809	4.502	10.656	7.103	7.420
Chandra et al. (3)	0.767	0.901	0.813	0.827	7.569	6.680	7.630	7.293
Ma et al. (4)	0.743	0.872	0.773	0.796	4.690	6.120	10.400	7.070
Chen et al. (43)	0.733	0.888	0.808	0.810	4.643	5.505	8.140	6.096
Zhang et al. (32)	0.791	0.903	0.836	0.843	3.992	4.998	6.369	5.120
Ours	0.801	0.909	0.854	0.855	3.879	4.571	6.411	4.954

*Higher Dice scores indicate the better results, while lower Hausdorff95 scores indicate the better results*.

**Figure 5:**
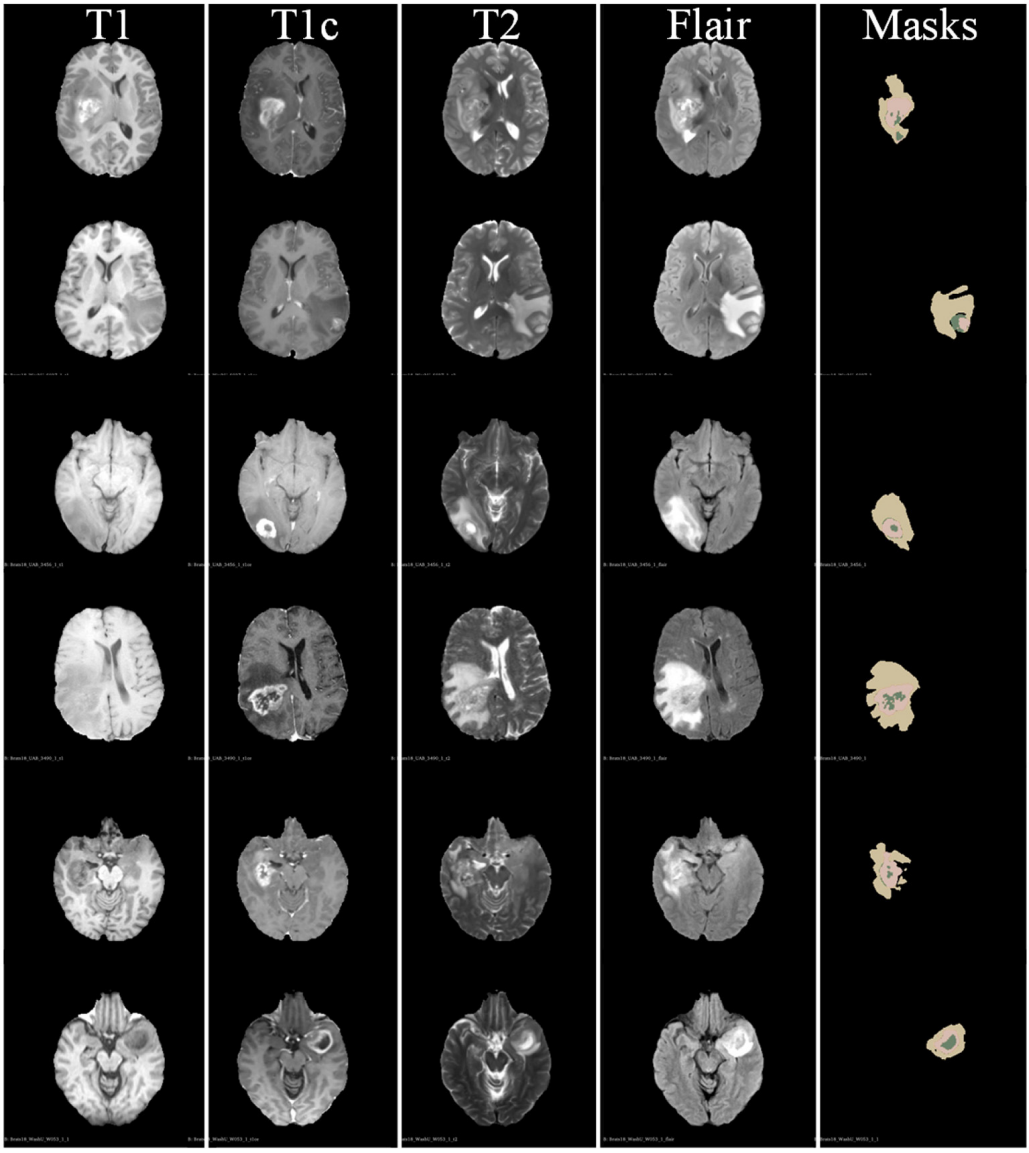
Some examples of segmentation results of our proposed brain tumor segmentation on BraTs 2018 dataset.

In Figure 5, we also show some examples of the brain tumor segmentation results for quantitative analysis. From Figure 5, we can observe that our method is more able to segment the details of the tumor areas, including TC areas, enhancing TC areas, and WT areas. The quantitative analysis results further illustrate the effectiveness of our proposed segmentation method.

## 7. Conclusion

In this work, we have proposed a novel attention-guided cross-modality feature learning framework for segmenting brain tumor areas from the multi-modality MRI data. Considering the fact that the texture and spatial position of normal organs in medical images have specific structural patterns, and deep neural networks can easily learn discriminant information from such regular patterns, we propose to mine the common normal patterns across the multi-modality data to captures the discriminative features between brain tumor areas and normal brain areas. The proposed learning framework consists of a cross-modality feature extracting module and an attention guided feature fusing module. By building a two-step feature interacting strategy, our proposed feature extracting module explores the multi-modalities interactive features that capture the rich information of the multi-modalities MRI data. The attention-guided feature fusing module encourages the feature extracting module to learn the structure patterns of the normal brain areas and aggregates the cross-modalities features in reasonable manner. Comprehensive experiments are conducted on BraTS 2018 benchmark, which demonstrate the effectiveness of our approach when compared to baseline models and state-of-the-art methods.

## Data Availability Statement

The dataset BraTs2018 for this study can be found in the MICCAI Brain Tumor Segmentation Challenge: http://braintumorsegmentation.org/.

## Author Contributions

BW and ZY contributed to the conception and design of the study. BW implemented the experiments and wrote the first draft of the manuscript. HP and LM contributed to clinical experience for the design of MRI segmentation model. All authors contributed to the result analysis, manuscript revision, read, and approved the submitted version.

## Conflict of Interest

BW and JA are employed by company Beijing Jingzhen Medical Technology Ltd. The remaining authors declare that the research was conducted in the absence of any commercial or financial relationships that could be construed as a potential conflict of interest.
